# The complete plastome sequence of *Atractylodes macrocephala* (Asteraceae: Cardueae), an important medicinal plant in East Asia

**DOI:** 10.1080/23802359.2020.1719926

**Published:** 2020-01-31

**Authors:** Huixia Cai, Chuan Chen, Yichen Wang, Hongyi Wang

**Affiliations:** a Laboratory of Systematic and Evolutionary Botany and Biodiversity, College of Life Sciences, Zhejiang University, Hangzhou, China;; b Hangzhou Botanical Garden, Hangzhou Academy of Landscape Science, Hangzhou, China

**Keywords:** *Atractylodes macrocephala*, cultivation, plastome genome, Cardueae, Asteraceae

## Abstract

*Atractylodes macrocephala* is one of the most commonly used herbs in China, which is famous for its high medicinal value. In this study, we analyzed and characterized the complete plastome sequence of *A. macrocephala.* Sequence analysis indicated that the entire genome is 153,265 bp in length, consisting of a large single-copy (LSC, 84,311 bp) and a small single-copy (SSC, 18,674 bp) region separated by a pair of inverted repeat (IR) regions of 25,140 bp for each. The genome contains 107 unique genes, including 80 different protein-coding genes, 23 tRNA genes, and 4 rRNA genes. The overall GC content of the genome is 37.7%. The phylogenetic analysis revealed a monophyletic *Atractylodes* and Cardueae. This research reports the complete plastome genome of *Atractylodes macrocephala*, which provides a better understanding of this important herb.

*Atractylodes macrocephala* Koidz. (Asteraceae: Cardueae) is an out-crossing perennial herb distributed on grasslands and forests of 600–2800 m in Jiangxi, Zhejiang, Sichuan, and Guizhou Provinces, China. It is endemic to China, and it was one of the first to be brought into cultivation as Chinese medicine (Zou 2010; Chen et al. 2018). Due to its medicinal values, *Atractylodes macrocephala* was introduced to Japan in the eighteenth century. The dried rhizome of *A. macrocephala* are used medicinally in traditional herbal remedies, called *Atractylodes macrocephala* Rhizoma (commonly referred to as ‘Baizhu’ in Chinese, ‘Byakujutsu’ in Japanese) (Shiba et al. 2006; Shi et al. 2012). Because of the over-exploitation and habitat destruction, natural population size of *A. macrocephala* have decreased and the wild herbs are under threat of extinction in several locations (Zou 2010; Zheng et al. 2012). An analysis of plastome information of *A. macrocephala* would provide abundant genetic information to identify, utilize and breed *A. macrocephala*.

Silica-gel dried leaves of *A. macrocephala* were collected from Mt. Tianmu, Zhejiang Province, China. Voucher specimen (*Li Zheng Z110905-2*) was deposited in the Herbarium of Zhejiang Univeristy (HZU). Total genomic DNA was extracted using the modified CTAB method (Murray and Thompson 1980). DNA was sheared to construct short-insert paired-end library in accordance with the Illumina HiSeq 2500 platform with read length of 150 bp in Beijing Genomics Institute (Wuhan, China). After filtering and error-correcting, the complete plastome sequence was assembled via NOVOPlasty (Dierckxsens et al. 2016). The plastome sequence of *A. chinensis* (NC_037484) was selected as a reference. The entire genome annotation was corrected with Geneious Prime v2019.2.1 (Kearse et al. 2012) following description in Liu et al. (2017) and Liu et al. (2018), and accomplished through the online program Dual Organellar Genome Annotator (DOGMA; Wyman et al. 2004), in addition, the circulare gene maps was generated by the OrganellarGenomeDRAW tool (OGDRAW) following by manual modification (Lohse et al. 2013). The complete plastome sequence of *A. macrocephala* was registered into GenBank with the accession number MN866906.

The whole plastome sequence of *A. macrocephala* is 153,265 bp in length with a typical quadripartite structure comprising an LSC region of 84,311 bp and an SSC region of 18,674 bp separated by a pair of IR regions of 25,140 bp. The overall GC content of the plastome is 37.7%, the GC contents of the LSC, SSC, and IR regions, are 35.8%, 31.6%, and 43.2%, respectively, which is similar to the plastomes from other Cardueae species. The plastome contains a total of 107 genes, 80 protein-coding genes, 23 tRNA genes and 4 rRNA genes were predicted. Among them, 18 genes (including 8 protein-coding genes, 6 tRNA genes and 4 rRNA genes) are located within the inverted repeat regions, therefore, occur as duplicates. Of the 107 distinct genes, 12 protein-coding genes held a single intron and two (*ycf3, clpP*) possessed two introns.

The phylogeny of Cardueae was reconstructed based on the multiple alignment of 33 plastome sequences, *Lactuca sativa* and *Taraxacum officinale* were chosen to be outgroups . The best model of nucleotide substitution was GTR + I + G, which was determined by the AIC in jModelTest2 on XSEDE on the CIPRES Science Gateway website (Miller et al. 2010). Maximum likelihood analysis (ML tree) was conducted using RAxML (Figure 1).

**Figure 1.**
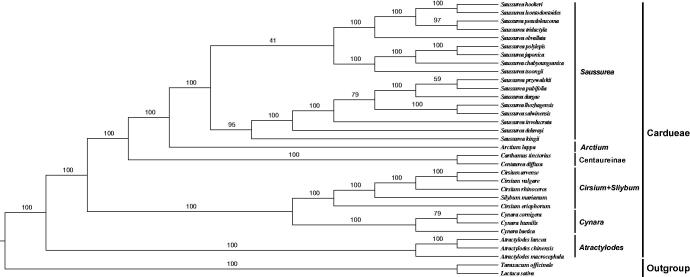
Phylogenetic tree using maximum likelihood (ML) based on plastomes of Cardueae species with two outgroups. Numbers near the nodes represent ML bootstrap values. Accession numbers: *Arctium lappa* NC_042724, *Atractylodes chinensis* NC_037484, *Atractylodes lancea* NC_037483, *Carthamus tinctorius* NC_030783, *Centaurea diffusa* NC_024286, *Cirsium arvense* NC_036965, *Cirsium eriophorum* NC_036966, *Cirsium rhinoceros* NC_044423, *Cirsium vulgare* NC_036967, *Cynara baetica* NC_028005, *Cynara cornigera* NC_028006, *Cynara humilis* NC_027113, *Saussurea chabyoungsanica* NC_036677, *Saussurea delavayi* NC_044733, *Saussurea durgae* NC_044735, *Saussurea hookeri* NC_044739, *Saussurea involucrate* NC_029465, *Saussurea japonica* NC_044738, *Saussurea kingie* NC_044736, *Saussurea leontodontoides* NC_044734, *Saussurea lhozhagensis* NC_044729, *Saussurea obvallata* NC_044726, *Saussurea polylepis* NC_036490, *Saussurea przewalskii* NC_044732, *Saussurea pseudoleucoma* NC_044728, *Saussurea pubifolia* NC_044727, *Saussurea salwinensis* NC_044731, *Saussurea tridactyla* NC_044730, *Saussurea tsoongii* NC_044737, *Silybum marianum* KT267161; *Lactuca sativa* AP007232, *Taraxacum officinale* KU361241 (Outgroup).
